# *In Vivo* Imaging of Macrophages during the Early-Stages of Abdominal Aortic Aneurysm Using High Resolution MRI in ApoE^−/−^ Mice

**DOI:** 10.1371/journal.pone.0033523

**Published:** 2012-03-20

**Authors:** Yuyu Yao, Yuanyuan Wang, Yi Zhang, Yefei Li, Zulong Sheng, Song Wen, Genshan Ma, Naifeng Liu, Fang Fang, Gao-Jun Teng

**Affiliations:** 1 Department of Cardiology, Zhongda Hospital, Medical School of Southeast University, Nanjing, Jiangsu, China; 2 Jiangsu Key Lab of Molecular and Function Imaging, Department of Radiology, Zhongda Hospital, Medical School of Southeast University, Nanjing, China; Universität Würzburg, Germany

## Abstract

**Background:**

Angiotensin II (ANG II) promotes vascular inflammation and induces abdominal aortic aneurysm (AAA) in hyperlipidemic apolipoprotein E knock-out (apoE^−/−^) mice. The aim of the present study was to detect macrophage activities in an ANG II-induced early-stage AAA model using superparamagnetic iron oxide (SPIO) as a marker.

**Methodology/Principal Findings:**

Twenty-six male apoE^−/−^ mice received saline or ANG II (1000 or 500 ng/kg/min) infusion for 14 days. All animals underwent MRI scanning following administration of SPIO with the exception of three mice in the 1000 ng ANG II group, which were scanned without SPIO administration. MR imaging was performed using black-blood T2 to proton density -weighted multi-spin multi-echo sequence. *In vivo* MRI measurement of SPIO uptake and abdominal aortic diameter were obtained. Prussian blue, CD68,α-SMC and MAC3 immunohistological stains were used for the detection of SPIO, macrophages and smooth muscle cells. ANG II infusion with 1000 ng/kg/min induced AAA in all of the apoE^−/−^ mice. ANG II infusion exhibited significantly higher degrees of SPIO uptake, which was detected using MRI as a distinct loss of signal intensity. The contrast-to-noise ratio value decreased in proportion to an increase in the number of iron-laden macrophages in the aneurysm. The aneurysmal vessel wall in both groups of ANG II treated mice contained more iron-positive macrophages than saline-treated mice. However, the presence of cells capable of phagocytosing haemosiderin in mural thrombi also induced low-signal-intensities *via* MRI imaging.

**Conclusions/Significance:**

SPIO is taken up by macrophages in the shoulder and the outer layer of AAA. This alters the MRI signaling properties and can be used in imaging inflammation associated with AAA. It is important to compare images of the aorta before and after SPIO injection.

## Introduction

Abdominal aortic aneurysm (AAA) is a major cause of mortality in the elderly population due to an increased risk of rupture [1]. Predicting the risk of rupture in AAA is clinically challenging [2]. Therefore, it is important to develop new biological markers and non-invasive techniques to detect the early development of AAA at a risk of rupture.

AAA is characterized by tissue degeneration, infiltration of inflammatory cells and subsequent dilation of the vessel [3]. Angiotensin II (ANG II) has been reported to accelerate atherosclerosis and induce aneurysms in hyperlipidemic apolipoprotein E deficient (apoE^−/−^) mice [4]. Histological studies have shown that the infiltration of macrophages and lymphocytes into the aorta occurs during the first days of ANG II infusion in apoE^−/−^mice [5]. Macrophages are involved in aneurysm growth and rupture in both animal models and patients [6], [7].

High-resolution MRI has emerged as the leading non-invasive *in vivo* imaging modality for atherosclerotic plaque characterization [8]. MRI has been used to assess AAA morphometry in mice *in vivo* [9]. Although aneurysm diameter and the expansion rate of the AAA are the criteria used to predict development and risk of rupture of an aneurysm, small aneurysms may also rupture [10]. Noninvasive detection of macrophages using MRI can contribute to the identification of aneurysms which are at an increased risk of growth or rupture [11]. In atherosclerosis, using superparamagnetic iron oxide (SPIO) or ultra-small SPIO (USPIO) as markers of activated monocytes/macrophages have been evaluated both experimentally and clinically [12], [13]. USPIO (mean diameter 20–30 nm) uptake has been used to detect macrophages within the walls of human AAA and murine models of AAA [7], [14]. SPIO (Resovist, Schering, Berlin, Germany) are larger nanoparticles (mean particle diameter 57 nm) than USPIO particles [15]. An *in vitro* study showed that SPIO underwent higher macrophage uptake compared with USPIO [16]. SPIO has been used to histologically quantify macrophage recruitment in atherosclerotic lesions in the apoE^−/−^ mouse following cytokine treatment [17]. So far, detection of macrophage activity using SPIO in MRI in a mouse model of early-stage AAA induced by Ang II has not yet been reported.

The aim of the present study was to perform an evaluation of SPIO as an *in vivo* imaging marker of macrophage phagocytic activities during the early-stages of ANG II-induced aortic aneurysm in apoE^−/−^ mice.

## Results

### Physiology, Lipid and Cytokine Profiles of AngII-Infused apoE^−/−^ Mice

**Figure 1:**
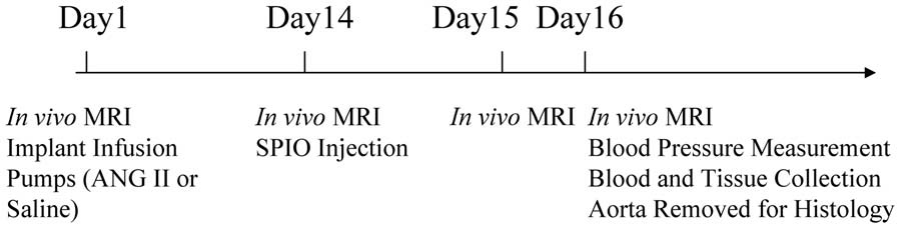
Experimental study design. Saline or ANG II were administrated *via* an osmotic minipump at day 1. Resovist (1 mmol/kg iron) was administered on Day 14 following ANG II infusion. Animals were imaged on Days 1, 14, 15 and 16 individually. Blood pressure was assessed on Day 16 prior to sacrifice. Tissue and blood were harvested on the completion of imaging on Day 16.

We followed the experimental study design as shown in Figure 1. Two animals died in the 1000 ng group due to the rupture of the thoracic aorta. No other mice in any experimental groups died during the course of the experiment. Of the mice in the 500 ng group, six developed suprarenal AAAs detected by MRI, the four mice without AAA were excluded from further analyses. AAA incidence was significantly different in ANG II-infused apoE^−/−^ mice administered in high and low doses (100% *vs.* 60% respectively). Aortic diameters of the group were increased compared with measurements at the baseline prior to ANGII infusion (Table 1). In the 1000 ng group, infusion of ANG II significantly increased aortic diameters (Table 1). Aortic diameters of saline-infused mice and control mice were not significantly different compared with measurements prior to infusion.

**Table 1:** Serum lipids and cytokines were measured in apoE^−/−^ mice after 14 days of Saline or Ang II administration.

	Control	Saline	ANGII 500 ng/kg/min	ANG II 1000 ng/kg/min
Body weight (g)	26.5±2.4	29.9±2.6	28.0±2.3	28.0±1.2
Mean blood Pressure (mmHg)	88.8±7.7	94.3±15	110.9±11	120.8±6.3*
Cholesterol (mg/dl)	103.9±35	696.7±114	641.3±114	656.4±91
Triglycerides ( mg/dl)	53.2±4.2	122.9±24.9	107.8±20.8*	114.8±14.4*
MCP-1 (pg/ml)	16.6±7.2	32.4±6.2	64.8±9.2*	84±14*
Lumen diameter (mm)	0.98±0.1	0.92±0.2	1.38±0.1*	1.82±0.2*

Data are presented as mean± SEM.

* *P*<0.05 *vs* Saline.

In apoE^−/−^ mice infused with ANG II (500 or 1,000 ng/kg/min), the mean arterial pressure was elevated within 14 days of infusion, but no significant effects on body weight, total serum cholesterol, or triglycerides concentrations were observed (Table 1).

MCP-1, a macrophage chemotactic factor, was examined in serum samples obtained from all animals at the end of study. There was a significant elevation in MCP-1 in the 500 or 1,000 ng ANG II groups (64.8±9.2 or 84±14 pg/mL) compared to the saline group (32.4±6.2 pg/mL, *P*<0.05).

### *In Vivo* MRI and Fe Analyses

**Figure 2:**
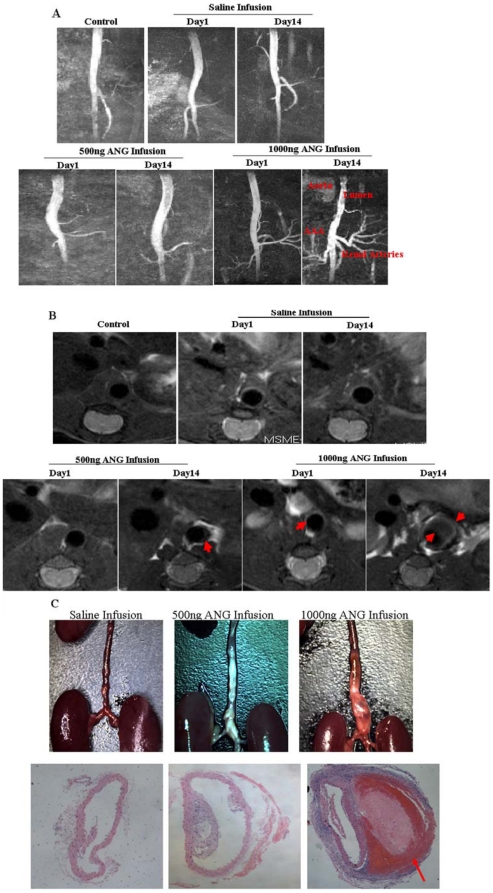
Identification of AAA in apoE^−/−^ mouse by *in vivo* MRI. (A) Representative abdominal aorta tree images in mice acquired at 7 T using 3D-FLASH sequence. The longitudinal view shows the characteristic dilatation of the abdominal aorta (B) *In vivo* image of a transverse section at baseline and following ANG II infusion using MSME Proton-density/T2-weighted sequences. Abdominal aorta with an aneurysm reveals a stenosed lumen after 14 days of ANG II infusion, an AAA has formed, resulted in a stenosed lumen and remodeled adventitia. Red arrows indicate aortic aneurysms. (C) The upper panel shows representative longitudinal views of the directly dissected abdominal aorta, the lower panel shows representative photomicrographs of aortic cross sections (H&E staining,10×). HE staining confirmed vessel expansion and dissection of the aorta in the 1000 ng/kg/min ANG II infusion group, medial destruction led to localized dissection and an aneurysm with intramural hemorrhage. Red arrows indicate intramural hemorrhage.

Aneurysms were clearly visualized on MRI in both groups of ANG II treated mice. Dilation of the abdominal aorta was identified in the suprarenal abdomen in these mice. None of the saline-treated apoE^−/−^ mice developed an aneurysm. The aneurysm was seen as a region of inhomogeneous low signal intensity compared with the bright signal in the aortic wall on T2-weighted (T2WI) MRI. Narrowing of the vessel lumen due to a large volume of mural thrombus was also noted (Fig. 2A, B).

On 3D FLASH MRI images, the longitudinal view showed an example of the characteristic dilatation of the abdominal aorta in the suprarenal region in ANG II treated mice (Fig. 2A).

Fig. 2B illustrates an example of MRI image of mice aortic dimensions at day 14. There is a 43.8% increase from the pretreatment baseline in mean aortic diameter in the 500 ng group with infusion of subpressor doses of ANG II. With a higher ANG II dose, apoE^−/−^mice at 14 days with an infusion of 1000 ng/min/kg ANG II had a 98% increase in suprarenal aortic diameters from pretreatment baseline. Quantitative comparison of the aortic diameters measured by MRI and measurement of the maximal width of excised aortas are shown in Fig. 2C. There was a statistically significant correlation between the MRI and histology measurements (r = 0.966, *P*<0.01).

**Figure 3:**
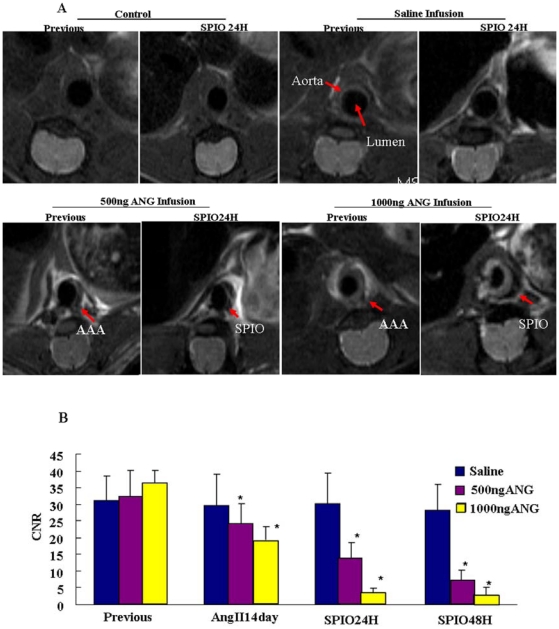
Representative example of T2WI of the abdominal aorta in apoE^−/−^ mice before and after SPIO injection. (A) The abdominal aorta before SPIO contrast agent administration and the same segment of the abdominal aorta 24 h post-SPIO administration. Red arrows indicate aortic aneurysms. (B) MRI signal in the abdominal aorta of apoE^−/−^ mice after injection of contrast agents. Values are expressed as mean ± SEM (n = 6, *P<0.05 vs. Previous & ANG II 14D).

Fig. 3A showed transverse slices at the level of the suprarenal abdominal aorta in representative mice before and 24-hours after SPIO administration. Significant MRI signal loss on T2WI sequences was observed in the suprarenal abdominal aorta of both groups of mice treated with ANG II infusion following SPIO administration (Fig. 3A); minimal MR signal loss was observed in the saline infusion group. In WT mice, no significant MRI signal changes in the arterial vessel wall were observed after administration of SPIO at any time points tested. The mean pre-SPIO contrast-to-noise ratio (CNR) of the 500 ng ANG II, 1000 ng ANG II, and saline groups in the abdominal wall at the level of the suprarenal abdominal aorta were 24.6±6.1, 18.7±4.1 and 29.9±9.4 respectively. Following SPIO injection, a significant reduction in CNR was observed in both 500 ng and 1000 ng ANG II groups (13.6±4.9, 3.4±1.5, *P*<0.001, n = 6), compared with the saline or WT controls (28.8±5.9, 22.2±5, n = 6) (Fig. 3B).

**Figure 4:**
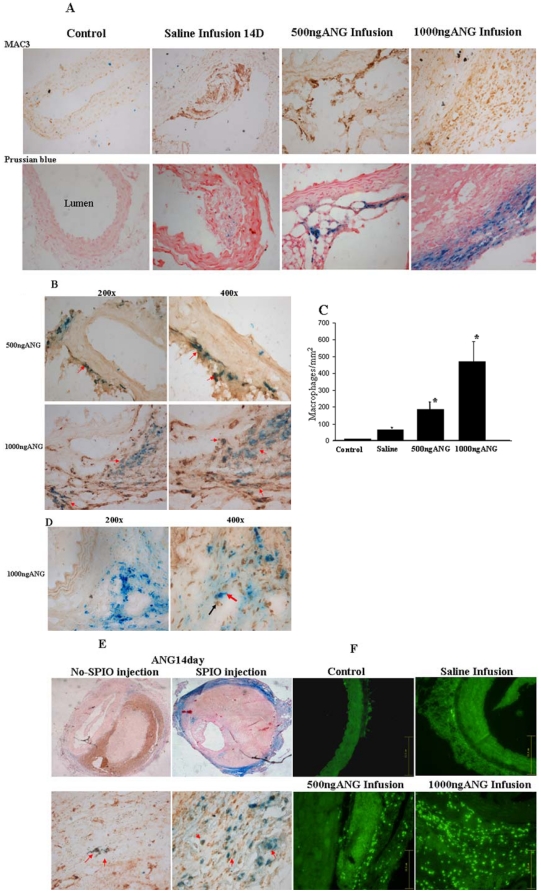
Representative histological sections of the abdominal aorta. (A) Upper panel: immunohistochemical analyses of aortic aneurysms, lesion area stained positive for Mac-3 (200×). Lower panel: the corresponding area of Prussian-blue staining demonstrated localization of iron positive staining (200×). (B) Double staining of CD68 positive macrophages (brown) and iron particles (blue) was performed. Upper panel is the 500 ng ANGII group, and lower panel is the 1000 ng ANGII group. Double staining confirmed that the high dose ANGII caused macrophage accumulation. (C). Quantitative analyses of Mac-3-positive cells. (D). Double staining of α-SMA positive smooth muscle cell (brown) and iron particles (blue) was performed. Red arrow indicated Prussian-blue positive cell, black arrow indicated SMC. SMC/Prussian-blue staining did not exhibit colocalization. (E). In the 1000 ng/kg/min ANG II-induced group without SPIO injection, Prussian blue-stained hemosiderin (blue) is shown in macrophages (brown) of the inner adventitia (left). Corresponding section of the SPIO injection group, with numerous Prussian blue-stained macrophages in the adventitia (right). (F). ANG II induced MMP activity. Representative images of MMP-9 immunostaining in the AAA area (200×).

Macrophages were observed entering the aneurysm from the shoulder and adventitial areas in the ANG II–induced mice (Fig. 4A upper panel). Mac-3 positive stained cells in five high-power fields were counted manually, showing significantly increased numbers of MAC-3-positive macrophages in the aneurysm in the two ANG II groups compared with the saline group (182.2±46.3, 472±118.7 *vs.* 61.3±9.3/mm2, n = 6, *P*<0.01). As shown in Fig. 4A, Prussian blue-stained iron was present in the macrophages of both ANG II-induced groups. Double staining for macrophages and iron showed significantly increased double staining cells in both ANGII-induced groups compared with the saline-treated group (140.2±19.7, 281±19.9 *vs.* 5.9±3.7/mm2, n = 6, *P*<0.01). In the saline infusion group, minimal scattered iron particle deposits were observed in the intima of the aortic wall (Fig. 4A). No iron deposits were seen in aortas of the wild-type control mice. To confirm MAC-3 immunohistological analyses data, we used anti-CD68 monoclonal antibody (a tissue macrophage marker). We found high levels of CD68 (+) monocyte and macrophage accumulation in the shoulder and adventitial. CD68 and Prussian blue-stained iron double positive cells were confirmed in two ANGII-infusion groups (Fig. 4B). The analysis showed that there was a strong correlation between macrophage content and *in vivo* CNR measurements yielding a correlation coefficient of 0.96 with a *P*<0.05. As shown in figure 4D, double staining of iron and α-smooth muscle actin (SMA, immunohistochemistry) confirmed that there was no apparent colocalization of the Prussian blue stain with smooth muscle cells in the shoulder area. This indicates considerable T2-weighted effects within the aneurysm at 48 hours following intravenous administration of SPIO where iron was phagocytosed by macrophages.

Immunohistochemistry revealed a marked upregulation of MMP-9 expression in the suprarenal aorta of ANG II-induced mice as compared to the aortic tissue of mice infused with saline (As shown in Fig. 4F). MMP-9 expression was colocalized with macrophage-like cells in the outer adventitia of aneurysm.

### Hemosiderin in Macrophages in the group without injection of SPIO

Compared with measurements at baseline before ANG II infusion (34.1±6.5, n = 11), MRI signals at aneurysm areas were significantly decreased in the ANG II-induced groups (18.7±4.1, n = 6, *P*<0.01). In the 1000 ng/kg/min ANG II-induced group with mural thrombus, Prussian blue-stained hemosiderin was observed in macrophages of the inner adventitia in three mice without SPIO injection (Fig. 4E).

## Materials and Methods

### Animal Model and Experimental Protocol

Animal care and experimental protocols were approved by the Southeast University Committee on Animal Care (approval ID: SYXK-2009.2981). Thirty-six week old apoE^−/−^ male mice, and background strain C57BL/6J mice, were provided by the Animal Center of Peking University Health Science Center (Beijing, China). Mice were fed with a standard lab chow. Mice were housed individually and were allowed free access to chow.

Animals were assigned to four groups: saline group received a saline infusion (n = 6), the 500 ng group received an ANG II infusion delivered at 500 ng/kg/min (n = 10), the 1000 ng group at 1000 ng/kg/min (n = 10), and C57BL/6 mice were used as the control group in the study (n = 5). ANG II (Sigma, St Louis, MO, USA) infusions were administered subcutaneously using osmotic mini pumps (Alzet, Model 1002) for 14 days at either 500 ng/kg/min (500 ng group) or 1000 ng/kg/min (1000 ng group). Mice were anesthetized with isoflurane gas during implantation and removal of osmotic mini pumps. Mini pumps were implanted subcutaneously in the mid-scapular region of the mice at day 1, and removed on day 14.

Fourteen days following ANG II or saline treatment, all animals underwent MRI scanning and administration of Resovist (1 mmol/kg iron, diluted with saline, three minutes) *via* the tail vein except the three mice in the 1000 ng group which were given only an ANG II infusion. At days 15 and 16, *in vivo* MRI was performed for each animal. Following *in vivo* MR examination, direct blood pressures were measured, whole blood was collected for determination of plasma lipids and cytokines. The abdominal aortic trunk was dissected and excised for histological analyses. A threshold of 1.22 mm for suprarenal aortic diameter was used as evidence of an incidence of aneurysm formation [18].

### *In Vivo* MRI Studies

MRI scanning was performed at baseline prior to ANG II or saline administration, and at the 14th day following ANG II infusion and days 1 and 2 after administration of SPIO.

All scanning was performed on a micro-MR animal scanner (7.0T Bruker PharmaScans, Bruker Biospin, Ettlingen, Germany). Each mouse was induced and maintained under isoflurane anesthesia (2%) in medical-grade air and monitored using the small animal instrument monitoring and gating system for respiration rate (reduced respiratory rate to 40 breath/min). All animals were placed in the supine position.

For MRI angiography, the abdominal aortic trees were imaged using a three-dimensional fast low-angle shot (3D-FLASH) sequence (repetition time (TR) was 15 ms, echo time (TE) was 2.5 ms. The field of view (FOV) was 3.5 cm×3.5 cm, and a 256×256×128 matrix was employed, yielding an voxel resolution 137×137×156 µm; flip angle (FA) was 20°; 1 excitations).

The suprarenal abdominal aorta was identified on the scout view of the coronal images. Eighteen contiguous, 1000-µm thick axial slices spanning from the abdominal aorta between the diaphragm and the renal artery were acquired using a spin echo sequence. MRI images were obtained using black-blood T2 to proton density (PD)-weighted multi-spin multi-echo (MSME) sequence and a dedicated mouse volume coil. Imaging parameters were as follows: TR 1206.9 ms, TE 12.8/34.2 ms, FOV 2.5 cm, FA was 180°, matrix size 256×256, and in-plane resolution 141×141×800 µm^3^; slice thickness of 1 mm, and four excitations. The imaging acquisition time was 30 minutes per animal. Fat suppression was performed for proton density and T2-weighted imaging. Coronal T1-weighted (MSME; TE 15 ms, TR 664 ms, 1 mm slice thickness, matrix 256×256, field of view (FOV) 2.5 cm, eight averages, 18 coronal slices, scan time 25 minutes) was acquired at exactly the same position.

Images were analyzed using the ParaVision software (PV5.0, Bruker, Germany). Continuous imaging on slices (n>5) allowed us to follow the CNR in the abdominal aorta over time. Signal intensities were measured by manually drawing a region of interest (ROI) within the wall of the suprarenal abdominal aorta and the vessel lumen. CNR was calculated for the signal intensity changes in similar sized regions of interest placed in the aortic lumen as well as within the vessel wall using the following equation: CNR = (wall signal−blood signal)/(standard deviation of the muscle signal).

### Blood Pressure Measurements

Following MRI acquisition on day 16, blood pressure was measured as previously described [19]. The common carotid artery was catheterized using a 2.5 French micro-manometer (Millar, Instruments, Houston, TX) by advancement into the aortic arches. Mean arterial pressure (MAP) was analyzed using Chart for Windows (Version 4) (AD Instruments).

### Blood Lipid and Vascular Studies

After blood collection, lethal doses of pentobarbital (120 mg/kg, IP) were administered. The collected blood was centrifuged at 1500 rpm for 10 minutes, and serum was separated and collected. Total cholesterol and triglyceride levels were assayed using diagnostic kits.

Monocyte chemotactic protein-1 (MCP-1) was measured in serum using an ELISA kit (Pierce Chemical Co. Rockford, IL).

### Morphometric Analyses

At the end of the procedure, the abdominal aorta was fixed in 4% paraformaldehyde and embedded. Serial sections of the abdominal aorta were cut at 3-mm intervals matching corresponding MR images. Transverse sections in 5 µm thickness were cut and stained with hematoxylin-eosin (HE). Each image was digitized with a camera and analyzed using a microscope. Prussian blue and MAC-3 (BD Biosciences Pharmingen, San Diego, CA), stains were used for detection of SPIO particles and macrophages, respectively. Sections were subjected to immunohistological analyses of matrix metalloproteinase 9 (MMP-9, BD Biosciences). To verify SPIO uptake by macrophages, serial sections were dually stained for Prussian blue iron and MAC-3. At least 10 sections were stained per mouse and quantification was performed blindly.

For CD68 immunohistochemical analyses, the abdominal aortas were collected from sacrificed mice, immediately placed in embedding medium (OCT compound), and rapidly frozen using liquid nitrogen. Frozen sections were prepared on a Leica CM1950 Cryostat (Leica Instruments, Heidelberger, Germany). Rat Anti-Mouse CD68, a tissue macrophage marker (clone FA-11; BioLegend, San Diego, USA), was used (1∶100) to access the deposition of macrophages in the abdominal aorta. For colocalization of SPIO particles, transverse sections were subjected to immunohistological analysis of macrophages (CD68), smooth muscle cells (rabbit against α-SMC, Abcam, Cambridge, UK), SPIO uptake was evaluated by Prussian blue staining on the same section.

### Statistical Analyses

Data was compared among experimental groups using ANOVA followed by Fisher's protected least-significant differences (PLSD). Data was expressed as mean ± standard error of the mean (SEM). Differences were considered statistically significant at a value of P<0.05. A Pearson correlation coefficient analysis was calculated to describe the relationship between MR and histological measurements.

## Discussion

This study demonstrates that the non-invasive assessment of SPIO contrast agent uptake *in vivo* using high field MRI can be used as a marker of macrophage activity during the early stages of ANG II-induced AAA in apoE^−/−^mice. There was a strong correlation between MR T2WI signal susceptibility by SPIO and macrophage infiltration within the aneurysm wall. However, endogenous iron in the thrombi and hemosiderin in the vessel wall from hemorrhages in arterial dissections can also be visualized using high resolution MR.

MRI can provide anatomical, structural and functional characterization of the arterial wall [20]. In this study, the 7.0T experimental MRI scanner offered adequate anatomic resolution for vascular morphology and signal characteristics, along with the location and size of the aneurysm. Adventitial remodeling and media rupture of the aneurysm were observed at 14 days following continuous ANG II infusion. Although in *vivo* MRI imaging correlated well with the pathologic features of the disease, it did not have enough resolution to visualize the location of the medial wall rupture, partially due to the 1 mm slice thickness [9].

**Figure 5:**
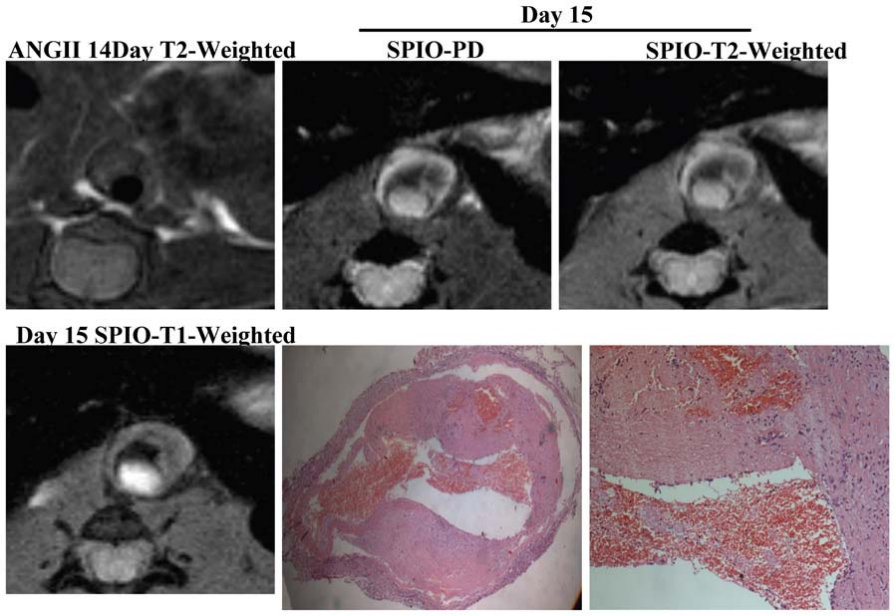
Acute thrombus formation. Compared with 14 days of ANG II infusion, MRI at day 15 showed acute artery occlusive and mural thrombi (red arrow hyperintense on T1-weighted images). HE showed fresh thrombus with platelets, inflammatory cells, and fibrin mesh.

Interestingly, the presence of acute thrombus formation was observed in one mouse in the 1000 ng group, as shown in Fig. 5, resulting in occlusion of the abdominal aorta. Mural thrombus formation appeared hyperintense on T1-weighted images on MRI at day 15. Histological examination found fresh thrombi with platelets, inflammatory cells, and fibrin mesh. Therefore, high-resolution MRI can not only provide anatomical information, such as the location, caliber and length, of an aneurysm, but also depict pathophysiological changes associated with the aneurysm, such as inflammatory reactions and mural thrombosis.

Research has demonstrated that assessment of USPIO uptake with MRI can be used to detect focal hotspots of inflammation in asymptomatic AAA [7]. Noninvasive imaging of macrophages may, therefore, yield valuable information about the pathogenesis of arterial aneurysm disease and predict the risk of aneurysm expansion and rupture [21], [11]. Previous studies have evaluated the use of intravenous USPIO and SPIO as MRI contrast agents for imaging macrophage activity in animal models of atherosclerosis and in human AAA [12], [21].

AAA almost always relates to atherosclerosis, which is essentially a chronic inflammatory process, but the pathophysiology of AAA is quite different from that of non-aneurysmal atherosclerotic plaques. In AAA, inflammation is mostly confined to the media and adventitia of the aorta, whereas in atherosclerotic plaques, the inflammatory reaction is seen primarily in the intima [22]. Atherosclerosis and AAAs display distinct histological features during initiation and progression, but have the common feature of macrophage infiltration throughout the progression of both disease processes [23].

Resovist [9] is the smallest of SPIO commercially available in the authors' country. SPIO particles are quickly phagocytosed by macrophages in the body, which can be visualized using MRI as areas of signal loss caused by MRI susceptibility on T2WI. The dose of SPIO administered in the present study was determined based upon the previous report which was almost 10-fold greater than that used in clinical medicine [17]. This was well tolerated in mice in our experimental conditions. SPIO can escape nonspecific uptake by the reticular-endothelial system (RES), which is engulfed by macrophages within vessels. Our results showed that the iron was mostly present within the shoulder and outer layer of the aneurysm vessel wall, and hypointense images on T2WI are associated with increased SPIO-laden macrophage. Most of the iron-laden macrophages were present in the region adjacent to the site of the external elastic lamina, although these areas were free of monocyte infiltration before the administration of ANG II and SPIO. Our results showed macrophage accumulation at the medial disruption areas, which was consistent with Daugherty's group who showed that there is macrophage accumulation in the media in response to AngII administration [4]. However, it is unclear as to how the SPIO particles ultimately reach the aortic wall. It is assumed that SPIO migrates across the interendothelial junction of the vasa vasorum into the interstitium and is then engulfed by macrophages at sites of inflammation [24]. Nevertheless, it remains unclear whether SPIO is phagocytosed while macrophages/monocytes are in the circulation. This study confirmed the location of iron-labeled cells in the aneurysm vessel wall through dual staining with Prussian blue and MAC-3 immunohistochemistry. Since Mac-3 is a marker of lysosomale activity associated with phagocytosis activity, we also used a tissue macrophage marker CD68 to confirm Prussian blue and macrophage double staining. Besides, controversy appears in the literature regarding the specific type of cell within the plaques that takes up the iron particles. Pande et al. showed that macrophages were the dominant cells for USPIO uptake; but some endothelial cells and smooth muscle cells were occasionally also noted to contain iron oxide particles [25]. Trivedi et al. found Prussian blue staining appeared to colocalize to macrophages in the shoulder regions of the plaque and no colocalization with smooth muscle and/or endothelial cell [24]. In the present study, double staining illustrates that SPIO colocalized with macrophages, but not with smooth muscles cells. These results demonstrate that iron-positive cells can be identified as macrophages.

It was observed that the SPIO-laden macrophages, which are abundant in the adventitial of the ANG II infusion groups, were minimal in atherosclerotic plaques in the saline-infusion group. Therefore, SPIO appears not suitable for the detection of plaques presumably because of its relatively large size which results in reduced transendothelial passage, tissue penetration, and easy uptake by the RES [26].

In a recently published article, SPIO-enhanced MRI visualizes leukocyte phagocytic activities in AAA patients [27]. This important clinical study shows that MRI imaging allows *in vivo* demonstration of SPIO uptake mainly localized at the luminal interfaces of the thrombi in high-risk AAAs. The uptake correlated with the abundance of leukocytes in the vessel luminal surface. While our study supports other published data by demonstrating that SPIO particles can be successfully used to monitor phagocytic activities in an AAA model of apoE^−/−^mice, we also showed few Prussian-blue positive cells in the inner adventitia in three mice of the group with high dose ANG II infusion without SPIO administration. This is important as it can be hypothesized that iron in these cells may be free iron released from the lysed red blood cell during intramural hemorrhage. It has been demonstrated that ANG II-induced mice have diffuse accumulation of macrophages in the media and adventitia as well as in foci of hemorrhages and thrombi [6]. Therefore, aneurysms with an acute intramural or mural thrombotic hemorrhage may cause the complicated MRI signals. It is known that fresh intraplaque hemorrhages produce isointensity or hypointensity on T2W/PD images [28]. Based on the findings in this study, reconsideration may need to be given to the interpretation of findings published by Nchimi A et al [27], as the iron observed in the Prussian-blue-positive leukocytes may partially originate from RBC-degradation products. It is important to perform a comparative analysis both pre- and post-MR images following intravenous injection of SPIO [29]. Furthermore, our study confirmed imaging of iron phagocytic activity could be enhanced by injections of SPIO [30].

To determine the origin of iron in Prussian-blue-positive macrophages, we further analyzed the SPIO-laden macrophages dependent on two points. We found that the number of Prussian blue-positive cells was much less in the mice without SPIO injection compared to the ones with SPIO injections. Therefore, imaging of iron phagocytic activity could be enhanced using intravenous injection of SPIO. Furthermore, the locations of hemosiderin-laden macrophages in non-SPIO injection group are associated with hemorrhages in the inner adventitia (Fig. 4E). These results suggest the presence of endogenous iron from intramural hemorrhages.

This study focused on early inflammatory events in an ANG II-induced AAA in a mouse model. Following 14 days of ANG II infusion, activated macrophages released cytokines and proteolytic enzymes. MMP-9 has also been found in the aneurysm vessel wall which primarily colocalized with infiltrated macrophages. Since AAA progression and eventual aortic rupture depends on the activity of macrophage-derived MMP-9 [31], developing an *in vivo* imaging approach for MMPs may provide an alternative for visualization of inflammation associated with AAA. This method offers a method to indirectly assess plaque macrophage content and macrophage activity.

In summary, this study demonstrates that imaging of macrophage activity in the vessel wall with SPIO provides a valuable tool for studying aneurysm biology. But signal loss on T2WI in the aneurysm vessel wall is also partially influenced by endogenous hemosiderin iron from intramural hemorrhages and thrombi. Noninvasinve visualization of SPIO-laden macrophages within an aneurysm may provide physiological information other than size in assessing the risk of acute AAA rupture.
